# On the consistency of relative facts

**DOI:** 10.1007/s13194-023-00551-8

**Published:** 2023-11-22

**Authors:** Eric G. Cavalcanti, Andrea Di Biagio, Carlo Rovelli

**Affiliations:** 1 Centre for Quantum Dynamics, Griffith University, Gold Coast, QLD 4222 Yugambeh Country Australia; 2 Institute for Quantum Optics and Quantum Information (IQOQI) Vienna, Austrian Academy of Sciences, Boltzmanngasse 3, A-1090 Vienna, Austria; 3 Basic Research Community for Physics e.V., Mariannenstraße 89, Leipzig, Germany; 4 CPT, Aix-Marseille University,Université de Toulon and CNRS, Luminy, Marseille, France; 5 Department of Philosophy and Rotman Institute of Philosophy, Western University, London ON, Canada; 6 Perimeter Institute, 31 Caroline Street N, Waterloo ON, Canada

**Keywords:** Quantum foundations, Relational quantum mechanics, Wigner’s friend paradox, Relative events

## Abstract

Lawrence et al. have presented an argument purporting to show that “relative facts do not exist” and, consequently, “Relational Quantum Mechanics is incompatible with quantum mechanics”. The argument is based on a GHZ-like contradiction between constraints satisfied by measurement outcomes in an extended Wigner’s friend scenario. Here we present a strengthened version of the argument, and show why, contrary to the claim by Lawrence et al., these arguments do not contradict the consistency of a theory of relative facts. Rather, considering this argument helps clarify how one should *not* think about a theory of relative facts, like RQM.

In Lawrence et al. (2023), Lawrence, Markiewicz, and Żukowski present an argument meant to show that “relative facts do not exist” and “Relational Quantum Mechanics (RQM) (Rovelli, 1996, 2018; Di Biagio & Rovelli, 2021) is incompatible with Quantum Mechanics”. Here we show why their conclusion is not warranted. We also present a strengthened version of the argument and argue that, although these arguments do not establish the inconsistency of relative facts, they nonetheless help clarify how one should *not* think about a theory of relative facts, like RQM.

The authors consider an extended Wigner’s friend version of a GHZ-type scenario. A system *S* formed by three qubits $(S_1,S_2,S_3)$ is prepared in a GHZ state (Greenberger et al. , 1990):1A triple of systems $(A_1,A_2,A_3)$, considered as observers, respectively measure a fixed observable for each of the three qubits and obtains outcomes $(\mathcal {{\mathcal A}}_1, {{\mathcal A}}_2, {{\mathcal A}}_3)$, where $i=1,2,3$. Subsequently, a second triple of observer systems $(B_1,B_2,B_3)$ respectively measures one observable for each pair of systems $(S_i, A_i)$ and obtains outcomes $({{\mathcal B}}_1, {{\mathcal B}}_2, {{\mathcal B}}_3)$.

Let us emphasise that, although the notation of Lawrence et al. (2023) does not make this clear, in RQM these outcomes have a value relative to each observer being considered, but not necessarily relative to every observer. In other words, they are relative events.

The observables are chosen in Lawrence et al. (2023) to parallel the proof of the GHZ theorem (Greenberger et al. , 1990) against the existence of local hidden variables. The authors of Lawrence et al. (2023) claim that the resulting set of six quantities $\{{\mathcal A}_1,{\mathcal A}_2,{\mathcal A}_3,{\mathcal B}_1,{\mathcal B}_2,{\mathcal B}_3\}$ must satisfy the four incompatible GHZ constraints

$$\begin{aligned} (\textrm{i})&:&\ \ \ \ {{\mathcal B}}_1{{\mathcal B}}_2{{\mathcal B}}_3=1, \\ (\textrm{ii})&:&\ \ \ \ {{\mathcal B}}_1{{\mathcal A}}_2{{\mathcal A}}_3 = -1, \\ (\textrm{iii})&:&\ \ \ \ {{\mathcal A}}_1{{\mathcal B}}_2{{\mathcal A}}_3 = -1, \\ (\textrm{iv})&:&\ \ \ \ {{\mathcal A}}_1{{\mathcal A}}_2{{\mathcal B}}_3 = -1. \end{aligned}$$

They conclude that this is an argument against the existence of the relative facts that RQM takes as its main ingredient.

Let us analyse this argument in detail. The authors of Lawrence et al. (2023) argue that each of the above measurements can be described as a unitary interaction between the system being measured and the corresponding observer system (what they call an “RQM-measurement”). This is in agreement with RQM. However, in RQM, quantum states are only interpreted as *relative* states in the sense of Everett (1957), and therefore any such unitary evolution is relative to a specific observer. In Lawrence et al. (2023), the observer from whose perspective the unitary description is given is not made explicit. However, in RQM a state of a system is always a state relative to another system. Here we consider for concreteness the unitary description to be relative to an observer *W* (“Wigner”) external to all of the systems considered above.

Using a notation only slightly different from that of Lawrence et al. (2023), the measurement of the Pauli *Y* observable of $S_m$ by $A_m$ can be described as unitary $\hat{U}_{{SA}_m}$ such that, when $S_m$ is prepared in a *Y*-eigenstate $|l^y\rangle _{S_m}$ ($l\in \{\pm 1\}$) and $A_m$ is initially in a “ready” state $|R\rangle _{A_m}$, we have

2 $$\begin{aligned} \hat{U}_{{SA}_m} \left( |l^y\rangle _{S_m} |R\rangle _{A_m} \right) =|l^y\rangle _{S_m} |l^y\rangle _{A_m}\,. \end{aligned}$$

Lawrence et al. (2023) consider then an entangling measurement by $B_m$ on the joint system $S_m\otimes A_m$. For simplicity of exposition, here (following Bong et al. (2020)) we consider instead a measurement that consists of first applying the inverse unitary $\hat{U}_{{SA}_m}^\dagger$, and then $B_m$ proceeding to measure the system $S_m$ directly on the Pauli *X* basis. This procedure leads to the same statistics.

Using this, Wigner describes the measurement by $B_m$ as a unitary interaction $\hat{U}_{{SAB}_m} = \hat{U}_{{SB}_m}\hat{U}_{{SA}_m}^\dagger$, where

3 $$\begin{aligned} \hat{U}_{{SB}_m} \left( |l^x\rangle _{S_m} |R\rangle _{B_m} \right) =|l^x\rangle _{S_m} |l^x\rangle _{B_m}\,. \end{aligned}$$

The sequence of all measurements then can be represented from Wigner’s perspective as follows,4where we have used5Now let us consider the constraints (i)-(iv) above, starting with (i). The authors of Lawrence et al. (2023) describe the composite system $B=B_1 \otimes B_2\otimes B_3$ as a single observer “*B*”, and similarly for $A=A_1\otimes A_2 \otimes A_3$. But this is not necessarily coherent with RQM.

Let us first consider the three systems $B_i$ as separate observers. The outcome ${\mathcal B}_i$ has a value relative to observer $B_m$, but can we say that the product of these outcomes should satisfy (i)? In RQM, as quoted in Lawrence et al. (2023), “it is meaningless to compare events relative to different systems, unless this is done relative to a (possibly third) system” and “comparisons can only be made by a (quantum-mechanical) interaction”. Thus, before the observers $B_m$ interact among themselves, or with a further observer, the constraint (i) has no meaning in RQM.

Let us then consider an interaction with Wigner, who measures each system $B_m$ on its “pointer basis”—that is, the basis $|l^x\rangle _{B_m}$ above, obtaining outcome ${\mathcal B}_m^W$. It is easy to show that the statistics for the product of these three measurements should correspond to the statistics for the product of three Pauli *X* measurements on the initial GHZ state. We can represent this process diagramatically as follows6This satisfies

$$\begin{aligned} (\mathrm {i'}) : \ \ \ \ {{\mathcal B}}_1^W{{\mathcal B}}_2^W{{\mathcal B}}_3^W=1. \end{aligned}$$

But this is not constraint (i), and in RQM we cannot infer constraint (i) from this constraint.

Similarly, constraint (ii) is not meaningful in RQM except relative to an observer that evaluates it. Let us then consider the situation where Wigner measures $B_1$ as above, but this time measures systems $A_2$ and $A_3$ on their pointer bases before $B_2$ and $B_3$ do their measurements, obtaining outcomes $({\mathcal B}_1^W,{\mathcal A}_2^W, {\mathcal A}_3^W)$:7The statistics for the product of these three measurements correspond to the statistics for the product of Pauli measurements $X_1Y_2Y_3$ on the initial GHZ state, thus satisfying

$$\begin{aligned} (\mathrm {ii'}) : \ \ \ \ {{\mathcal B}}_1^W{{\mathcal A}}_2^W{{\mathcal A}}_3^W=-1. \end{aligned}$$

But as before, this is not constraint (ii), and in RQM we cannot infer constraint (ii) from this constraint. A similar analysis holds for constraints (iii) and (iv).

We therefore conclude that *none* of the constraints (i)-(iv) hold a priori in RQM, contrary to the claim by Lawrence et al. (2023). Only one of the four constraints (i’)-(iv’) can hold relative to Wigner, with the rest being meaningless. Each constraint corresponds to a different context, where Wigner makes a different triple of measurements. To be clear, Wigner can meaningfully predict, before choosing his measurements, that the following constraints hold as expectation values, if those measurements are performed by him:

$$\begin{aligned} (\mathrm {i'''})&:&\ \ \ \ \langle {\hat{X}}_1{\hat{X}}_2{\hat{X}}_3\rangle _W=1,\\ (\mathrm {ii'''})&:&\ \ \ \ \langle {\hat{X}}_1{\hat{Y}}_2{\hat{Y}}_3\rangle _W = -1, \\ (\mathrm {iii'''})&:&\ \ \ \ \langle {\hat{Y}}_1{\hat{X}}_2{\hat{Y}}_3\rangle _W = -1, \\ (\mathrm {iv'''})&:&\ \ \ \ \langle {\hat{Y}}_1{\hat{Y}}_2{\hat{X}}_3\rangle _W = -1. \end{aligned}$$

But when we write ${\mathcal B}_1^W$, we are referring to a measurement outcome actually obtained by Wigner in a particular run. Of course, this value only exists (relative to Wigner) in the runs where Wigner performs that measurement. Following Peres (1978), “unperformed measurements have no outcomes”.

On the other hand, one may ask: isn’t it the case that all the six quantities $\{{\mathcal A}_1,{\mathcal A}_2,{\mathcal A}_3,{\mathcal B}_1,{\mathcal B}_2,{\mathcal B}_3\}$ refer to *performed* measurements? Don’t they all have a value in each run of the experiment, then? And if so, shouldn’t those values obey the constraints (i)-(iv)?

This is a subtle point. The key is that although those measurements are all performed *by some observer* in each run of the experiment, there is no observer relative to whom they all take co-existing values. One may invoke the “cross-perspective link” (Adlam & Rovelli, 2022) to conclude that *if* Wigner performs one of the six measurements above (say if he observes outcome ${\mathcal B}_1^W$), *then* he can conclude that ${\mathcal B}_1^W={\mathcal B}_1$. If he observes the triple ${\mathcal B}_1^W{\mathcal B}_2^W{\mathcal B}_3^W$, he should obtain values compatible with (i’), and therefore in that case he could conclude that constraint (i) holds for the values observed by $B_1$, $B_2$ and $B_3$. One cannot however simply *define* an observer $B = B_1 \otimes B_2 \otimes B_3$ relative to which constraint (i) holds, if there is no interaction involving those three systems after their measurements take place. A similar argument can be made to conclude that *if* Wigner performs the measurements corresponding to one of the other constraints (ii’)-(iv’), *then* the corresponding constraint (ii)-(iv) holds. But this does not allow us to infer that all four constraints must be a priori satisfied.

We close with a general philosophical consideration. As repeatedly stated in the original papers, RQM does not necessarily require a commitment to a specific philosophy. However, it does highlight the cost that quantum mechanics puts upon different metaphysical options. The scenario analysed here is a good example. Different philosophical attitudes can be considered, with respect to the metaphysical status of the list $\{{\mathcal A}_1,{\mathcal A}_2,{\mathcal A}_3,{\mathcal B}_1,{\mathcal B}_2,{\mathcal B}_3\}$. One possibility is the choice of declaring it part of reality, even if no observer has simultaneous access to all of those values (see Adlam & Rovelli, 2022). The “cost” of this option is that reality, so defined, violates a number of features that we commonly expect it to respect (Brukner , 2018; Bong et al. , 2020; Cavalcanti & Wiseman, 2021; Haddara & Cavalcanti, 2023) – an assignment of values to all of those quantities amounts to an assumption of “Absoluteness of Observed Events”, implying the rejection of at least one of the other premises of various no-go theorems (Bong et al. , 2020; Cavalcanti & Wiseman, 2021; Haddara & Cavalcanti, 2023). Alternatively, one may choose a more radical relationalism, and assume that only assertions relative to a physical system are to be taken as meaningful statements about reality. In this case, the elements of the list are part of reality relative to each observer making those measurements, but the complete list is not part of reality, because there is no observer relative to which all of those observables take co-existing values.
